# Dual functions of a novel effector in the plant and pathogen arms race

**DOI:** 10.1007/s44154-023-00116-y

**Published:** 2023-08-18

**Authors:** Han Lu, Mingjun Gao

**Affiliations:** Ministry of Education Key Laboratory for Biodiversity Science and Ecological Engineering, National Observations and Research Station for Wetland Ecosystems of the Yangtze Estuary, Institute of Biodiversity Science and Institute of Eco-Chongming, School of Life Sciences, Fudan University, Shanghai, China

**Keywords:** *Ralstonia solanacearum*, Bacterial wilt, PehC, Immune response

## Abstract

*Ralstonia solanacearum* is a soil-borne bacterium that causes bacterial wilt disease in over 250 plant species. It has been identified as one of the top ten most serious plant pathogenic bacteria globally, causing significant crop yield loss every year. Despite its large impact on agricultural economics, the molecular mechanisms underlying plant defense against *Ralstonia* infection and by which *Ralstonia* grows within the plant xylem remain largely unexplored. In a recent article, Ke et al. discovered a distinct pathogen effector, which acted as an immune elicitor in plants but also played dual roles in compromising plant immune activation and increasing nutrient acquisition from the host plants for pathogen propagation.

## Main text

*Ralstonia solanacearum* is a gram-negative soil-borne pathogen that can infect a broad range of plants including tomato (*Solanum lycopersicum*) and potato (*Solanum tuberosum*) (Mansfield et al. 2012). After penetration into the root of the host plants, *Ralstonia solanacearum* quickly spreads to the aerial tissues through xylem vessels, leading to wilt disease (Genin and Denny 2012; Lowe-Power et al. 2018). However, the mechanisms by which the effectors of *Ralstonia* perturb host immunity, as well as how the pathogens acquire and utilize nutrients during infection, remain poorly understood.

To better control the *Ralstonia*, it is necessary to conduct in-depth exploration and research on its parasitism and infection mechanisms.

Plants have evolved a multi-layered immune system, including pathogen/microbe-associated molecular patterns (PAMPs or MAMPs)-triggered immunity (PTI) and effector-triggered immunity (ETI) (Jones and Dangl 2006). Plasma membrane-localized pattern recognition receptors (PRRs) sense the conserved PAMPs or MAMPs to initiate PTI. PRRs can also recognize damage-associated molecular patterns (DAMPs) from plants to activate DAMP-triggered immunity (DTI). DAMPs include pectin-derived oligogalacturonides (OGs), plant-induced peptides (PIP), as well as extracellular ATPs (Gust et al. 2017). *R. solanacearum* uses the Type II Secretion System to deliver effector proteins into the plant cells and hijacks the host cellular machinery to benefit pathogen growth. Various factors, referred to as *Ralstonia* injected proteins (Rips), contribute to *R. solanacearum* infection and virulence (Landry et al. 2020). A team led by Prof. Bo Li from Huazhong Agricultural University has been conducting in-depth research on the plant-*Ralstonia* interactions, aiming to unravel the intricate mechanisms involved in the pathogenesis of bacterial wilt and the activation of plant immune responses. Previously, they had shown that, in *Arabidopsis*, the type III effector RipAB interacts with the TGA transcription factors to attenuate immune gene expression (Qi et al. 2022). In a recent study, they have discovered a new strategy employed by *Ralstonia* to avoid plant immune activation (Ke et al. 2023). They found that in tomato the *Ralstonia* secreted PehC, which encodes an exo-polygalacturonase, could hydrolyze OGs produced from pectin to inhibit the DAMP-triggered immunity, whereas PehC itself was recognized by unknown host receptor proteins in tomato roots thus to exhibit elicitor activity. The resulting products of PehC-mediated hydrolysis of OGs, the GalA monomers, served as carbon resources for *Ralstonia* growth.

Ke et al. (2023) first examined the immune activities of the secreted proteins (SPs) from *Ralstonia* strain GMI1000. They found that inoculation of tomato roots with SPs led to increased extracellular pH, reactive oxygen species (ROS) burst, activation of mitogen-activated protein kinase (MAPK) cascades, and upregulation of immune genes, suggesting that SPs could trigger plant immune responses. To characterize the potential factors responsible for SP-mediated immune activation, they performed liquid chromatography-tandem mass spectrometry (LC–MS/MS) analysis and identified PehC as the causal candidate. This was further confirmed by *in planta* assays, in which the GST-PehC recombinant protein was found to induce MAPK activation, ROS burst, and callose deposition in tomato roots, but not in the leaves. In contrast, immune activation was not detected for PehA and PehB, indicating functional specificity of PehC.

Next, Ke et al. (2023) tested the biological significance of PehC-triggered immunity. Pretreatment of Moneymaker (susceptible) roots with PehC enhanced disease resistance, which was associated with increased expression of genes involved in defense and oxidation–reduction reactions, as revealed by RNA sequencing analysis. Notably, PehC triggered global transcriptional reprograming in a way similarly to flgII-28, the distinct MAMP from *Pseudomonas* specifically responsible for PTI in tomato. BAK1 and SERK4 are co-receptors of Receptor-Like Kinases (RLKs), and SOBIR1 is a co-receptor of Receptor-Like Proteins (RLPs) (Liebrand et al. 2014). PehC-induced ROS burst and MAPK activation were greatly abolished in the *bak1-5 serk4-1* double mutant, while the *sobir1* mutation did not interfere with PehC activity, suggesting that the recognition of PehC was dependent on the presence of the co-receptors BAK1 and SERK4.

To reveal the functions of PehC in *Ralstonia* growth and infection, the authors generated a *pehC* deletion mutant (Δ*pehC*), a complementation strain (*ComPehC*), and an inactive variant (*ComH453A*). Surprisingly, mutation of *PehC* did not promote virulence. Through LC–MS analysis, they found that PehC could hydrolyze OGs, resulting in the release of the monosaccharide GalA. As OGs act as DAMPs in diverse plant systems (Ferrari et al. 2013), degradation of OGs by PehC would evade immune activation.

Considering that *Ralstonia* propagates in the vascular xylem, which is a nutrient-poor environment, Ke et al. (2023) speculated that the components of the cell wall polysaccharides degraded by bacterial PehC might be utilized by *Ralstonia* as a carbon source. In support of this hypothesis, the growth fitness of the Δ*pehC* mutant in tomato roots was reduced compared to that of the wild-type strain. LC–MS analysis of xylem sap collected from *Ralstonia* infected plants identified peaked accumulation of GalA. A polysaccharide feeding assay demonstrated that *Ralstonia* can utilize GalA as a carbon source for growth and reproduction. Therefore, GalA produced by PehC from OGs serves as an important carbon source for *Ralstonia* growth in the xylem during the early stages of infection.

In summary, Ke et al. provide evidence that PehC, a protein secreted by *Ralstonia* through type II secretion system, has specialized functions in host–pathogen interaction- it enhanced virulence by hydrolyzing pectin-derived OGs to evade OG-triggered DTI and the resulting GalA monomers serve as carbon source to promote nutrient acquisition and bacterial growth. The virulence strategy employed by *Ralstonia* represents a major advancement in the understanding of the ongoing evolutionary arms race between plants and pathogens. PehC exists in only a small group of bacteria, suggesting that it might emerge during the co-evolution with the host plants. The characterization of the cognate RLK-type receptor(s) of PehC would provide new insights into the molecular mechanisms of pathogen recognition and plant immune signaling.

## Data Availability

Not applicable.

## Abbreviations

PAMPs: Pathogen-associated molecular patterns
MAMPs: Microbe-associated molecular patterns
PTI: Pattern-triggered immunity
ETI: Effector-triggered immunity
DAMPs: Damage-associated molecular patterns
DTI: DAMP-triggered immunity
OGs: Oligogalacturonides
PIPs: Plant-induced peptides
SPs: Secreted proteins
T3SS: Type III Secretion System
LC–MS/MS: Liquid chromatography-tandem mass spectrometry
RLKs: Receptor-Like Kinases
RLPs: Receptor-Like Proteins
ROS: Reactive oxygen species
MAPK: Mitogen-activated protein kinase

## References

[CR1] Ferrari S, Savatin DV, Sicilia F, Gramegna G, Cervone F, De Lorenzo G (2013). Oligogalacturonides: plant damage-associated molecular patterns and regulators of growth and development. Front Plant Sci.

[CR2] Genin S, Denny TP (2012). Pathogenomics of the *Ralstonia solanacearum* species complex. Annu Rev Phytopathol.

[CR3] Gust AA, Pruitt R, Nürnberger T (2017). Sensing danger: key to activating plant immunity. Trends Plant Sci.

[CR4] Jones JDG, Dangl JL (2006). The plant immune system. Nature.

[CR5] Ke J, Zhu W, Yuan Y, Du X, Xu A, Zhang D, Cao S, Chen W, Lin Y, Xie J, Cheng J, Fu Y, Jiang D, Yu X, Li B (2023) Duality of immune recognition by tomato and virulence activity of the *Ralstonia solanacearum* exo-polygalacturonase PehC. Plant Cell koad098. 10.1093/plcell/koad098 10.1093/plcell/koad098 PMC10291029 36977631

[CR6] Landry D, González-Fuente M, Deslandes L, Peeters N (2020). The large, diverse, and robust arsenal of *Ralstonia solanacearum* type III effectors and their in planta functions. Mol Plant Pathol.

[CR7] Liebrand TWH, van den Burg HA, Joosten MHAJ (2014). Two for all: receptor-associated kinases SOBIR1 and BAK1. Trends Plant Sci.

[CR8] Lowe-Power TM, Khokhani D, Allen C (2018). How *Ralstonia solanacearum* exploits and thrives in the flowing plant xylem environment. Trends Microbiol.

[CR9] Mansfield J, Genin S, Magori S, Citovsky V, Sriariyanum M, Ronald P, Dow M, Verdier V, Beer SV, Machado MA, Toth I, Salmond G, Foster GD (2012). Top 10 plant pathogenic bacteria in molecular plant pathology. Mol Plant Pathol.

[CR10] Qi P, Huang M, Hu X, Zhang Y, Wang Y, Li P, Chen S, Zhang D, Cao S, Zhu W, Xie J, Cheng J, Fu Y, Jiang D, Yu X, Li B (2022). A *Ralstonia solanacearum* effector targets TGA transcription factors to subvert salicylic acid signaling. Plant Cell.

